# Long-term outcomes of prediction error after combined phacoemulsification and trabeculectomy in glaucoma patients

**DOI:** 10.1186/s12886-021-01824-7

**Published:** 2021-01-26

**Authors:** Yeon Soo Kang, Mi Sun Sung, Hwan Heo, Yong Sok Ji, Sang Woo Park

**Affiliations:** Department of Ophthalmology, Chonnam National University Medical School and Hospital, 42 Jebong-ro, Dong-gu, Gwangju, 61469 South Korea

**Keywords:** Phacotrabeculectomy, Prediction error, Anterior segment parameters, Medically uncontrolled glaucoma, Lens vault

## Abstract

**Background:**

To investigate long-term outcomes of prediction error after phacotrabeculectomy and to determine risk factors that may cause unstable prediction error after phacotrabeculectomy in glaucoma patients.

**Methods:**

A total 120 eyes of 120 patients who had underwent uncomplicated phacotrabeculectomy (combined group) or phacoemulsification (phaco-only group) were included. Best-corrected visual acuity (BCVA), intraocular pressure (IOP) were measured before and after surgery, and anterior segment parameters including anterior chamber depth (ACD), lens vault (LV), and anterior vault (AV) measured using anterior-segment optical coherence tomography were compared between the two groups. The mean absolute error (MAE) at 3, 6, 12, and 24 months postoperatively were compared. Risk factors associated with unstable prediction error (MAE ≥ 0.5) were investigated in the combined group.

**Results:**

In both groups, BCVA was improved and IOP was decreased significantly. MAE at 3, 6, 12, 24 months postoperatively were not significantly different between two groups. The risk factors for unstable prediction error after 12 months of phacotrabeculectomy were old age and LV. Whereas, the only factor predicting unstable prediction error after 24 months of phacotrabeculectomy was LV. The cut-off value of LV for predicting unstable refractive error analyzed by the ROC curve was 0.855 mm.

**Conclusions:**

Phacotrabeculectomy may be an effective treatment with stable long-term outcomes of prediction error similar to phacoemulsification in patients with glaucoma. However, elderly patients or patients with large LV may be predisposed to unstable prediction error after phacotrabeculectomy.

## Background

When planning the surgery of a patient with cataract and medically uncontrolled glaucoma, it becomes difficult to decide whether to perform trabeculectomy and phacoemulsification at the same time or to perform phacoemulsification after trabeculectomy. It has already been reported that axial length (AL) and anterior chamber depth (ACD) decrease [1–3] and keratometry changes [4–6] after trabeculectomy. Because these parameters play an important role in determining the intraocular lens (IOL) power [7–10], there is a possibility that postoperative prediction errors may increase if the IOL power calculation formula used for phacoemulsification is used when performing phacotrabeculectomy [11, 12].

If phacoemulsification is performed with uncontrolled intraocular pressure (IOP), surgery becomes difficult due to high posterior pressure [13]. Complications such as hypotony, hyphema, shallow anterior chamber may occur after phacotrabeculectomy [14, 15], and the frequency of complications is higher than that of phacoemulsification alone [16, 17]. Nevertheless, there are several advantages of phacotrabeculectomy such as improving visual acuity after surgery, minimizing postoperative IOP spikes, and reducing morbidity that can occur in two stage operations [18–20]. Since the success rate has been increased by the using of mitomycin C [21, 22], phacotrabeculectomy is widely performed in recent clinical trials.

As the calculations for IOL power improve due to the development of preoperative evaluations and surgical techniques, refractive errors are becoming more predictable [22, 23]. There is increasing interest in reducing prediction errors after phacotrabeculectomy because trabeculectomy can affect refractive errors after phacoemulsification. Recently, several studies focusing on outcomes of prediction error after phacotrabeculectomy have been published [11, 18, 22–25].

However, most of the previous studies examined short-term outcomes of prediction error less than 6 months, and no studies have investigated risk factors that can cause unstable prediction error after phacotrabeculectomy. Therefore, the present study aims to compare the long-term outcomes of prediction error of phacotrabeculectomy and phacoemulsification, and to determine the factors that predict unstable refractive error after phacotrabeculectomy.

## Methods

### Subjects

We retrospectively reviewed medical records of patients who had underwent uncomplicated phacotrabeculectomy (combined group) or uncomplicated phacoemulsification (phaco-only group) between September 2015 and December 2018. Patients underwent phacotrabeculectomy when IOP was not controlled even with the maximal tolerated medical treatment. Patients who were able to follow-up for more than 1 year were included, and exclusion criteria included prior keratorefractive surgery, ocular disease that may affect refractive errors except glaucoma and cataract, IOL in the ciliary sulcus or sclera fixation of IOL, and bleb needling after phacotrabeculectomy. If both eyes of a patient satisfied these criteria, one eye was chosen randomly. Ethical approval was obtained from the Chonnam National University Hospital Institutional Review Board, and the study protocol followed the guidelines of the Declaration of Helsinki.

### Data collection

All patients underwent preoperative evaluations including best-corrected visual acuity (BCVA), manifest refraction using autorefractor keratometer (ARK; Topcon KR-8900®, Topcon, Tokyo, Japan), IOP using Goldmann applanation tonometry, and slit-lamp examination. For comparison, BCVA was converted to logarithm of the minimum angle of resolution (LogMAR) and the refraction value was converted to spherical equivalent (SE) by adding spherical power to 1/2 of cylinder power. And 12 months after the surgery, BCVA and IOP were measured to investigate the effect of the two operations on visual acuity and IOP.

K-value using ARK and axial length (AL) using partial coherence interferometry (Lenstar®, Haag-Streit, Bern, Switzerland) were measured, and SRK-T formula was used to calculate IOL power and predicted refractive errors. Additionally, anterior segment parameters were measured by using anterior-segment optical coherence tomography (AS-OCT) device (Visante®, Carl Zeiss Meditec, Dublin, CA). A single examiner (S.W.P) selected the best images with no motion artifacts, good visibility of the scleral spur, and no image artifacts from the eyelids. And then, two independent examiners (Y.S.K and M.S.S) who were blinded to other clinical information analyzed images using custom software (Iridocorneal module, Carl Zeiss Meditec). Anterior chamber depth (ACD) was defined as the distance between the center of posterior corneal surface and anterior lens surface, lens vault (LV) was defined as the maximum perpendicular distance between the anterior lens surface and horizontal line connecting the two sclera spurs, and anterior vault (AV) was defined as the sum of ACD and LV [26].

### Surgical technique

Surgical procedures were performed by a single surgeon (S.W.P) under topical or retrobulbar anesthesia. Standard phacoemulsification was used to remove the cataract through a temporal 2.8 mm clear corneal incision. In all cases, Acrysof SN60WF (Alcon, Fort Worth, TX) IOL was implanted in the capsular bag.

For a phacotrabeculectomy, a fornix-based conjunctival flap and rectangular shaped half-thickness scleral flap were created. Phacoemulsification and IOL implantation were performed through clear corneal incision at a different site with trabeculectomy. A sponge soaked in 0.04% mitomycin C was placed under the conjunctiva and Tenon’s capsule on the sclera for 3–5 min according to the discretion of the surgeon and based on the patient characteristics. Sclerotomy and peripheral iridectomy were performed. Finally, the scleral flap was sutured with 2 interrupted sutures using 10–0 nylon and the conjunctiva was closed.

### Outcome measures

Mean absolute error (MAE) was defined as the absolute value of the difference between predicted refractive errors and postoperative refractive errors. MAE at 3, 6, 12, and 24 months postoperatively were compared between two groups. The combined group was further divided into two subgroups based on the prediction errors: stable prediction error (MAE < 0.5 diopters [D]) and unstable prediction error (MAE ≥ 0.5 D). Risk factors associated with unstable prediction error were investigated at 12 and 24 months postoperatively. The number of glaucoma medications used preoperatively and 12 and 24 months postoperatively were also compared to determine the IOP lowering effect of phacotrabeculectomy.

### Statistical analysis

SPSS version 26.0 (SPSS Inc., Chicago, IL) was used for all statistical analyses. Independent *t*-test or Mann-Whitney U test was used to compare the continuous data of the two groups, and paired *t*-test was used to compare the values before and after surgery in the same group. Chi-square test was used to compare categorical data, and logistic regression analysis was used to investigate risk factors that may cause unstable prediction error. Variables with a significance level at *P* < 0.1 in the univariable analysis were included in the multivariable analysis. The receiver operating characteristic (ROC) curve analysis was used to determine the optimal cut-off value of risk factor predicting unstable refractive error. The ability of the cut-off value to predict accurately is represented by the area under the curve (AUC). *P* values less than 0.05 were considered statistically significant.

## Results

A total 120 eyes of 120 patients were enrolled in this study, and 60 eyes were included in each group. The subject’s demographics and baseline characteristics of the included eyes are summarized in Table 1. Since patients in the combined group had medically uncontrolled glaucoma, the mean preoperative IOP was 29.60 ± 10.04 mmHg, which was significantly higher than those in phaco-only group (*P* < 0.001). ACD was significant smaller in the combined group than that of the phaco-only group (*P* = 0.018). There were no significant differences in age, sex, preoperative BCVA, AL, LV, and AV.

**Table 1:** Subject’s demographics and baseline characteristics of included eyes

Variables	Combined group (***n***=60)	Phaco-only group (***n=***60)	***P*** value
Age (years)	65.65 ± 11.02	67.83 ± 8.88	0.234 ^a^
Sex (male/female)	34 / 26	30 / 30	0.464 ^b^
Baseline BCVA (LogMAR)	0.67 ± 0.82	0.47 ± 0.58	0.124 ^a^
Baseline IOP (mmHg)	29.60 ± 10.04	14.52 ± 2.50	< 0.001 ^a^
AL (mm)	23.16 ± 1.04	23.26 ± 1.17	0.337 ^a^
ACD (mm)	2.25 ± 0.48	2.48 ± 0.56	0.018 ^a^
LV (mm)	0.82 ± 0.36	0.71 ± 0.38	0.090 ^a^
AV (mm)	3.06 ± 0.40	3.19 ± 0.38	0.089 ^a^

*BCVA* best-corrected visual acuity, *LogMAR* logarithm of the minimum angle of resolution, *IOP* intraocular pressure, *AL* axial length, *ACD* anterior chamber depth, *LV* lens vault, *AV* anterior vault

^a^Independent t-test for combined group and phaco-only group

^b^Chi-square test for combined group and phaco-only group

In both combined group and phaco-only group, BCVA was significantly improved (*P* = 0.001 and *P* < 0.001, respectively) and IOP was significantly decreased (*P* < 0.001 and *P* < 0.001, respectively) at 12 months postoperatively (Table 2). The phaco-only group tended to show better postoperative BCVA than the combined group, but there was no statistical significance (*P* = 0.065). Mean postoperative IOP of the combined group was 13.35 ± 3.12 mmHg, which remained stable until 12 months after phacotrabeculectomy. MAE at 3, 6, 12, and 24 months postoperatively were not significantly different between the two groups (*P* = 0.072, *P* = 0.117, *P* = 0.226, and *P* = 0.083, respectively) (Table 3). That is, outcomes of prediction error after phacotrabeculectomy were similar with that of the phacoemulsification alone until 24 months postoperatively.

**Table 2:** Visual outcomes and IOP reduction at 12 months postoperatively

Variables	Combined group (***n=***60)	Phaco-only group (***n=***60)	***P*** value ^**a**^
Baseline			
BCVA (LogMAR)	0.68 ± 0.82	0.47 ± 0.58	0.124
IOP (mmHg)	29.60 ± 10.04	14.52 ± 2.50	< 0.001
Postoperative			
BCVA (LogMAR)	0.48 ± 0.86	0.24 ± 0.56	0.065
IOP (mmHg)	13.35 ± 3.12	13.23 ± 2.20	0.814
***P*** **value** ^**b**^			
BCVA	0.001	< 0.001	
IOP	< 0.001	< 0.001	

*IOP* indicates intraocular pressure, *BCVA* best-corrected visual acuity, *LogMAR* logarithm of the minimum angle of resolution

^a^Independent *t*-test for combined group and phaco-only group

^b^Paired *t*-test for baseline and postoperative values

**Table 3:** Outcomes of prediction error of the combined group and phaco-only groups

Variables	Combined group (***n=***60)	Phaco-only group (***n=***60)	***P*** value ^**a**^
MAE at 3 months postoperatively (D)	0.69 ± 0.47	0.54 ± 0.47	0.072
MAE at 6 months postoperatively (D)	0.66 ± 0.60	0.52 ± 0.32	0.117
MAE at 12 months postoperatively (D)	0.59 ± 0.44	0.49 ± 0.43	0.226
MAE at 24 months postoperatively (D) ^b^	0.63 ± 0.44	0.47 ± 0.36	0.083

*MAE* mean absolute error, *D* diopters

^a^Independent *t*-test for combined group and phaco-only group

^b^Only forty-seven patients were included in each of groups at 24 months postoperatively

Table 4 showed characteristics of the stable and unstable prediction error subgroups in the combined group at 12 and 24 months postoperatively. Patients in the unstable subgroup were significantly older (*P* = 0.024) and had a larger LV (*P* = 0.038) and larger AV (*P* = 0.041) than those in the stable subgroup at 12 months postoperatively. And patients in the unstable subgroup had significantly shallower ACD (*P* = 0.040) and larger LV (*P* = 0.010) than those in the stable subgroup at 24 months postoperatively.

**Table 4:** Characteristics of the stable and unstable subgroups in combined group at 12 and 24 months postoperatively

Variables	12 months postoperatively			24 months postoperatively		
	Stable subgroup (***n=***30)	Unstable subgroup (***n=***30)	***P*** value	Stable subgroup (***n***=20)	Unstable subgroup (***n***=27)	***P*** value
Age (years)	62.47 ± 10.68	68.83 ± 10.58	0.024 ^a^	62.40 ± 11.77	66.40 ± 10.55	0.222 ^c^
Sex (male/female)	17 / 13	17 / 13	1.000 ^b^	12 / 8	16 / 11	0.599 ^b^
Type of glaucoma (ACG/OAG)	17 / 13	17 / 13	1.000 ^b^	9 / 11	17 / 10	0.177 ^b^
Baseline BCVA (LogMAR)	0.61 ± 0.82	0.74 ± 0.82	0.530 ^a^	0.87 ± 1.05	0.44 ± 0.39	0.197 ^c^
Postoperative BCVA (LogMAR)	0.40 ± 0.76	0.57 ± 0.95	0.449 ^a^	0.59 ± 10.7	0.36 ± 0.60	0.334 ^c^
Baseline IOP (mmHg)	31.87 ± 10.50	27.33 ± 9.18	0.080 ^a^	30.15 ± 9.23	29.37 ± 10.01	0.384 ^c^
Postoperative IOP (mmHg)	13.50 ± 2.67	13.20 ± 3.57	0.714 ^a^	13.75 ± 2.86	13.48 ± 3.41	0.312 ^c^
AL (mm)	23.11 ± 0.89	23.20 ± 1.19	0.735 ^a^	23.20 ± 0.94	23.22 ± 1.16	0.249 ^c^
ACD (mm)	2.23 ± 0.47	2.27 ± 0.50	0.492 ^a^	2.28 ± 0.46	2.08 ± 0.41	0.040 ^c^
LV (mm)	0.74 ± 0.31	0.89 ± 0.27	0.038 ^a^	0.70 ± 0.35	0.94 ± 0.21	0.010 ^c^
AV (mm)	2.96 ± 0.27	3.17 ± 0.47	0.041 ^a^	2.99 ± 0.26	3.02 ± 0.45	0.418 ^c^

*ACG* angle closure glaucoma, *OAG* open angle glaucoma, *BCVA* best-corrected visual acuity, *LogMAR* logarithm of the minimum angle of resolution, *IOP*, intraocular pressure, *AL* axial length, *ACD* anterior chamber depth, *LV* lens vault, *AV* anterior vault

^a^Independent t-test for stable subgroup and unstable subgroup

^b^Chi-square test for stable subgroup and unstable subgroup

^c^Mann-Whitney U test for stable subgroup and unstable subgroup

The univariable analysis showed that old age (OR = 1.060, *P* = 0.031), large LV (OR = 6.838, *P* = 0.045), and large AV (OR = 4.873, *P* = 0.047) were associated with unstable prediction error at postoperative 12 months after combined surgery. The multivariable analysis showed that old age (OR = 1.069, *P* = 0.030) and large LV (OR = 5.687, *P* = 0.029) were risk factors of unstable prediction error at 12 months postoperatively (Table 5). At postoperative 24 months, univariable and multivariable analysis determined that only large LV (OR = 19.647, *P* = 0.024) was associated with unstable prediction error after combined surgery (Table 6).

**Table 5:** Factors associated with unstable prediction error in combined group at 12 months postoperatively

Variables	Univariable analysis			Multivariable analysis *		
	OR	95% CI	***P*** value ^**a**^	OR	95% CI	***P*** value ^**a**^
Age, per 1 year older	1.060	1.005–1.118	0.031	1.069	1.007–1.135	0.030
Male gender	1.000	0.360–2.777	1.000			
ACG	1.000	0.360–2.777	1.000			
Baseline BCVA, per 1 increase	1.229	0.651–2.321	0.524			
Baseline IOP, per 1 mmHg increase	0.953	0.903–1.006	0.084	0.963	0.905–1.024	0.229
AL, per 1 mm increase	1.090	0.667–1.782	0.730			
ACD, per 1 mm increase	1.449	0.511–4.103	0.485			
LV, per 1 mm increase	6.838	1.043–44.809	0.045	5.687	1.195–27.064	0.029
AV, per 1 mm increase	4.873	1.210–19.625	0.047	6.231	0.755–51.425	0.089

*OR* odds ratio, *CI* confidence interval, *ACG* angle closure glaucoma, *BCVA* best-corrected visual acuity, *IOP* intraocular pressure, *AL* axial length, *ACD* anterior chamber depth, *LV* lens vault, *AV* anterior vault

^a^Logistic regression analysis

* Only variables with a *P* value of less than .10 in the univariable analysis were included in the multivariable model

**Table 6:** Factors associated with unstable prediction error in combined group at 24 months postoperatively

Variables	Univariable analysis			Multivariable analysis *		
	OR	95% CI	***P*** value ^**a**^	OR	95% CI	***P*** value ^**a**^
Age, per 1 year older	1.034	0.980–1.092	0.226			
Male gender	1.031	0.317–3.352	0.959			
ACG	2.078	0.640–6.744	0.223			
Baseline BCVA, per 1 increase	0.442	0.176–1.110	0.082	0.460	0.157–1.349	0.157
Baseline IOP, per 1 mmHg increase	0.991	0.933–1.054	0.781			
AL, per 1 mm increase	1.022	0.589–1.771	0.939			
ACD, per 1 mm increase	0.346	0.088–1.362	0.129			
LV, per 1 mm increase	21.309	1.813–250.393	0.015	19.647	1.469–262.802	0.024
AV, per 1 mm increase	1.260	0.270–5.878	0.769			

*OR* odds ratio, *CI* confidence interval, *ACG* angle closure glaucoma, *BCVA* best-corrected visual acuity, *IOP* intraocular pressure, *AL* axial length, *ACD* anterior chamber depth, *LV* lens vault, *AV* anterior vault

^a^Logistic regression analysis

* Only variables with a *P* value of less than .10 in the univariable analysis were included in the multivariable model

**Fig. 1:**
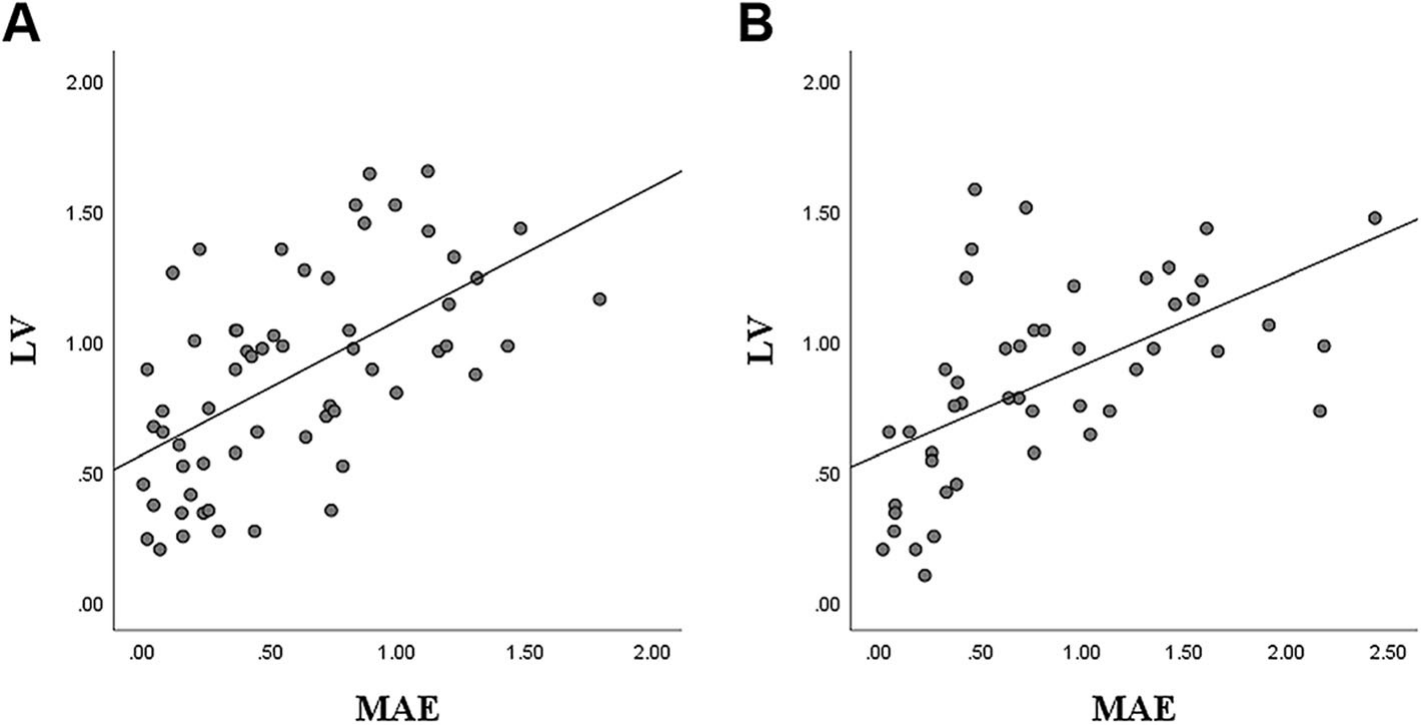
Scatter plot of LV and MAE at 12 months postoperatively (**a**) and 24 months postoperatively (**b**). LV = lens vault; MAE = mean absolute error

**Fig. 2:**
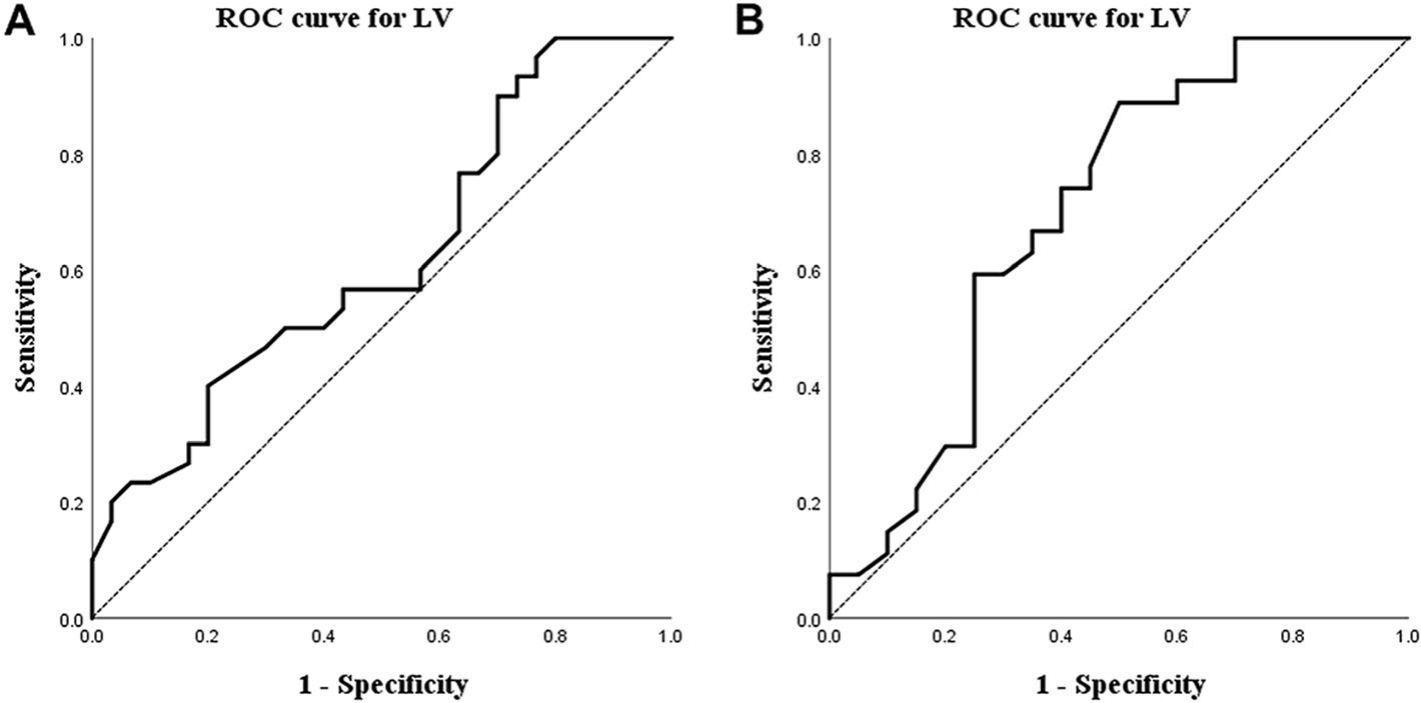
Graphs showing the ROC curve of LV for unstable prediction error at 12 months postoperatively (**a**) and at 24 months postoperatively (**b**). The AUC of (**a**) was 0.619 and the AUC of (**b**) was 0.689. The cut-off value of LV was 0.855 mm in both (**a**) and (**b**). ROC = receiver operating characteristic; LV = lens vault; AUC = area under the curve

The scatter plot of LV and MAE at 12 and 24 months postoperatively showed their relationships (Fig. 1). ROC curve analysis was performed to determine the optimal cut-off value of the LV that can predict unstable refractive errors (Fig. 2). The AUC of LV at 12 months postoperatively was 0.619 and the AUC of LV at 24 months postoperatively was 0.689. In both ROC curves, the cut-off value of LV was 0.855 mm.

The patients in the combined group used an average of 2.8 medications preoperatively. The needs for glaucoma medication were significantly reduced to average of 1.12 medications at 12 months (*P* < 0.001) and average of 1.27 medications at 24 months (*P* < 0.001) postoperatively.

## Discussion

Cataract surgery has the advantage of quick visual recovery and relatively low complications [27–29]. Since cataract surgery alone has an effect of reducing IOP in patients with angle closure glaucoma (ACG) [16, 17, 30] and open angle glaucoma (OAG) [31–33], phacoemulsification plays an important role in the treatment of glaucoma. In patients with ACG, phacoemulsification significantly deepens the ACD and resolves angle crowding. Because these changes are small in patients with OAG, the exact mechanism why IOP decreases after cataract surgery in patients with OAG remains controversial [29]. Some studies reported that both changes of angle configuration and trabecular meshwork or extracellular matrix remodeling are involved [27, 28]. In the current study, the fact that IOP significantly decreased postoperatively in the phaco-only group also supports previous studies.

Preoperatively, the ACD of the combined group was significantly shallower than that of the phaco-only group. This finding can be explained by the prevalence of ACG (34 eyes) in the combined group, whereas the phaco-only group had twelve patients with ACG. Previous studies reported that the refractive errors of patients with ACG were difficult to predict [34, 35], but our result showed relatively stable prediction error despite the large number of patients with ACG in the combined group.

Recently, the indications of phacotrabeculectomy are as follows: i) medically uncontrolled glaucoma, ii) tolerance of glaucoma medications, iii) postoperative IOP spikes may worsen visual field damage, iv) suspected compliance of glaucoma medications [15–17, 36]. Several studies reported that the IOP lowering effect of phacotrabeculectomy was superior to that of phacoemulsification alone in patients with glaucoma [36–38]. In the present study, the mean preoperative IOP of combined group was 29.60 ± 10.04 mmHg. It was significantly reduced to average of 13.35 ± 3.12 mmHg at 12 months postoperatively, and the postoperative need of glaucoma medications was significantly decreased. As in the previous studies, phacotrabeculectomy was found to be an effective treatment for lowering IOP.

In this study, we initially aimed to investigate prediction error after phacotrabeculectomy at 12 months postoperatively, the medical records of 120 patients in combined group and phaco-only group were analyzed. Among them, there were many patients who had medical records up to 2 years after the surgery, the data of 94 patients were additionally analyzed. And we found that there was no significant difference in prediction error between phacotrabeculectomy and phacoemulsification alone until 24 months after surgery. It means that phacotrabeculectomy was effective not only for IOP control but also for stable prediction error.

Previous studies reported that myopic shift occurs after phacotrabeculectomy compared to phacoemulsification alone [11, 23–25]. A decreased ACD after trabeculectomy causes a myopic shift, and a decreased AL after trabeculectomy causes a hyperopic shift, conversely. Some authors of these studies estimated that a decrease in ACD had a greater effect on refractive errors than a decrease in AL, leading to myopic shift. In the current study, since the prediction errors were compared using the absolute value of the difference of refractive errors, we could not analyze whether myopic shift or hyperopic shift occurred after phacotrabeculectomy.

Law et al. [24] reported that K-value was increased after phacotrabeculectomy. Changes of not only ACD and AL but also K-value affect prediction errors, and if K-value increases, hyperopic shift may occur. Since this study did not measure postoperative K-value, ACD, and AL, it was not possible to analyze which factors had more significant effect on prediction errors. However, we hypothesized that there was no significant difference in prediction errors between combined group and phaco-only group because the changes of ACD, AL, and K-value after phacotrabeculectomy had a global effect.

Most of the studies that focused the prediction errors of phacotrabeculectomy analyzed only short-term outcomes less than 6 months [11, 18, 22–25], and one study reported by Chung et al. [12] had a limitation that the follow-up period of the control group was average of 4.81 months. Therefore, the present study is clinically significant because we analyzed long-term outcomes of prediction error up to 24 months after phacotrabeculectomy.

Tzu et al. [39] reported that the risk factor for prediction errors in combined cataract and glaucoma surgery included old age. Though the follow-up time was less than 6 months, glaucoma drainage device surgery was included in the combined group, it remains a meaningful result. Old age has been associated with structural changes of scleral collagen fiber and changes of ACD [40, 41], therefore, some studies reported that age of patients may affect the prediction errors after cataract surgery [42, 43]. Our study also supports this result, as old age appeared to be a risk factor up to 12 months after phacotrabeculectomy. However, old age did not appear as a risk factor after 24 months of phacotrabeculectomy, which may be because of the relatively small number of patients.

Our group previously reported that one risk factor causing unstable prediction errors after cataract surgery in patients with glaucoma was large LV [44]. We speculated that large LV predispose to larger displacement of IOL position, resulting in unstable prediction error. In agreement with the previous report, in the current study, large LV was a risk factor that could cause unstable prediction errors up to 12 and 24 months after phacotrabeculectomy. Therefore, we suggest that LV plays an important role in predicting refractive errors after combined phacotrabeculectomy surgery in glaucoma patients as well as cataract surgery.

Ozaki et al. [45] reported that the LV of primary angle closure (PAC) patients was 1.034 mm on average, and that of normal people was 0.419 mm, and Hsia YC et al. [46] reported that LV of OAG patients was 0.55 mm on average. It has already been found that the increased LV is a risk factor of PAC and a predictive factor for refractive errors after cataract surgery in patients with glaucoma [44, 45], but there have been no studies analyzing the cut-off value of LV that can cause unstable prediction errors. We analyzed the long-term data after phacotrabeculectomy and found that a LV thickness of 0.855 or more was a risk factor for unstable prediction errors. Therefore, the surgeon should be very careful when operating patients with LV greater than 0.855 mm because unstable prediction errors can be obtained.

The current study has several limitations. First, we could not analyze which ocular parameters had a significant effect on refractive outcomes because some ocular parameters were not measured after surgery. Second, both patients with ACG and patients with OAG were included in the combined group, so the effect of angle status was not considered. Third, we did not consider digital massage or releasable suture removal that could affect refractive errors after phacotrabeculectomy. Forth, SRK-T formula was used instead of using the widely used Barrett or Haigis formulas, which is known to have high accuracy recently. Finally, we did not analyze the complications that could occur after phacotrabeculectomy because only patients without complication were included. In the future, larger and more long-term studies that consider these factors will be needed.

## Conclusions

In conclusion, phacotrabeculectomy is an effective treatment that significantly reduces IOP and decreases the use of glaucoma medications in patients with cataract and medically uncontrolled glaucoma. There was no significant difference in prediction errors compared to phacoemulsification alone, and patients who underwent phacotrabeculectomy could also get stable prediction errors. However, the operator should be careful phacotrabeculectomy may increase in elderly patients or patients with large LV.

## Data Availability

The datasets generated and/or analyzed during the current study are not publicly available to protect patient identify and confidentiality but are available from the corresponding author on reasonable request.

## Abbreviations

AL: Axial length
ACD: Anterior chamber depth
IOL: Intraocular lens
IOP: Intraocular pressure
BCVA: Best-corrected visual acuity
ARK: Automated keratorefractometer
LogMAR: Logarithm of the minimum angle of resolution
SE: Spherical equivalent
AS-OCT: Anterior segment optical coherence tomography
LV: Lens vault
AV: Anterior vault
MAE: Mean absolute error
ROC: Receiver operating characteristic
AUC: Area under the curve
ACG: Angle closure glaucoma
OAG: Open angle glaucoma
PAC: Primary angle closure
